# RECRUITING LGBT OLDER ADULTS FOR RESEARCH

**DOI:** 10.1093/geroni/igz038.2198

**Published:** 2019-11-08

**Authors:** Anyah Prasad, Alice Fisher, Michael Immel, Kathrin Boerner, Kamal Jethwani, Timothy hale, Amanda Centi

**Affiliations:** 1 University of Massachusetts Boston, Boston, Massachusetts, United States; 2 Osher Lifelong Learning Institute, Boston, Massachusetts, United States; 3 Connected Health Innovation – Partners Health Care, Boston, Massachusetts, United States

## Abstract

LGBT older adults constitute a rare population and so are methodologically difficult to recruit. Due to stigma, many of them may not disclose their sexual/gender identity, which makes it challenging for researchers to reach out to them. Due to history of discrimination, LGBT older adults may not trust researchers. The purpose of this presentation is to discuss strategies used to recruit LGBT older adults to a study on exploring the idea of an online senior center for LGBT older adults in Massachusetts. Building a rapport with community stakeholders, developing trust and having LGBT older adults themselves as part of the research team were important tools to help overcome these challenges. LGBT older adults are very diverse and focused efforts should be made to recruit them from various racial/ethnic backgrounds, rural areas; also, those who are not publicly open about their identity, and who are home bound due to restricted mobility.

